# miR-200b restrains EMT and aggressiveness and regulates matrix composition depending on ER status and signaling in mammary cancer

**DOI:** 10.1016/j.mbplus.2020.100024

**Published:** 2020-01-22

**Authors:** Zoi Piperigkou, Marco Franchi, Christoph Riethmüller, Martin Götte, Nikos K. Karamanos

**Affiliations:** a Biochemistry, Biochemical Analysis & Matrix Pathobiology Research Group, Laboratory of Biochemistry, Department of Chemistry, University of Patras, Patras, Greece; b Department for Life Quality Studies, University of Bologna, Rimini, Italy; c Centre for Nanotechnology, Serend-ip GmbH, Münster, Germany; d Department of Gynecology and Obstetrics, Münster University Hospital, Münster, Germany; e Foundation for Research and Technology-Hellas (FORTH)/Institute of Chemical Engineering Sciences (ICE-HT), Patras, Greece

**Keywords:** ECM, extracellular matrix, EGFR, epidermal growth factor receptor, EMT, epithelial-to-mesenchymal-transition, ER, estrogen receptor, Erk, extracellular signal-regulated kinase, GAG, glycosaminoglycan, GF, growth factor, HER2, human epidermal growth factor receptor 2, IGF-IR, insulin-like growth factor receptor type I, IL, interleukin, miRNA, microRNA, MMP, matrix metalloproteinase, PG, proteoglycan, PR, progesterone receptor, pre-miRNA, precursor miRNA, RISC, RNA-induced silencing complex, SERM, selective estrogen receptor modulator, TGFβ, transforming growth factor beta, Breast cancer, Estrogen receptors, Extracellular matrix, miRNAs, miR-200b

## Abstract

Secreted microRNAs (miRNAs) reside in a complex regulatory network with extracellular matrix (ECM) macromolecules, which affect cell-cell communication, therefore miRNA expression highlights its significance in several aspects of human diseases, including cancer. miRNA-mediated regulation of breast cancer has received considerable attention due to evidence that shows miRNAs to mediate estrogen receptor (ER) status, metastasis, chemoresistance and epithelial-to-mesenchymal transition (EMT). miR-200b is a pluripotent miRNA, which is inversely regulated by ERα and ERβ in mammary cancer. It has been identified as tumor suppressor and EMT inhibitor serving as a critical biomarker, as its expression in breast tumor determines the disease-free survival, thus highlighting its roles in breast cancer invasion and metastasis. The main goal of this study was to investigate the role of miR-200b in modulating the behavior of breast cancer cells with different ER status. We demonstrate that estrogen signaling through ERs reduces miR-200b expression levels in ERα-positive breast cancer cells. Moreover, miR-200b upregulation reduces the aggressive phenotype of ERβ-positive breast cancer cells by inhibiting cell invasiveness and motility, followed by ECM reorganization as well as cytoskeletal and morphological changes concluded from deep inspection of cell topography. Future investigation towards the mechanistic perspective of miR-200b effects in the behavior of aggressive mammary cancer cells appears rewarding in order to expand our understanding of miR-200b as a novel mediator beyond breast cancer diagnosis and pharmaceutical targeting.

## Highlights

• Estrogen receptors mediate miR-200b expression in mammary cancer cells.
• Lower miR-200b levels have been correlated with poor disease-free survival.
• miR-200b restrains the aggressiveness of MDA-MB-231 breast cancer cells.
• miR-200b overexpression demonstrates high-impact effects in ECM composition and signaling molecules.
• The novel role of miR-200b targeting in aggressive breast cancer is highlighted.

## Introduction

With approximately 1,676,600 new cases per year worldwide, breast cancer is the most frequent malignancy in women [1]. The expression status of steroid hormone receptors [estrogen receptor alpha/progesterone receptor (ERα/PR)], human epidermal growth factor receptor 2 (HER2), or the absence of these receptors (triple-negative) is an important diagnostic tool for patient stratification, and guides treatment options towards anti-hormonal therapy with selective estrogen receptor modulators (SERMs), such as tamoxifen for ERα-positive breast cancer, or HER2-targeting antibodies (Herceptin®) [2,3]. Even though there is a significant progress in targeted therapies and immunotherapy, breast cancer mortality is high, with approximately 521,900 annual deaths [1,4]. Breast cancer mortality is closely linked to the process of metastasis to vital organs [5]. Metastasis is promoted both by tumor cell-autonomous changes in cell motility and by close interactions of cancer cells with their extracellular and cellular microenvironment, which drive metastatic behavior [[4], [5], [6], [7]]. For instance, the upregulation of heparanase and matrix metalloproteinases (MMPs) promotes degradation of basement membranes and interstitial extracellular matrix (ECM) [6,8]. Constitutive signaling mutations and upregulation of chemokine, angiogenesis and growth factor receptors and their ligands, as well as remodeling processes that affect ECM stiffness, promote breast cancer cell motility, chemotaxis to distant sites and angiogenesis as a prerequisite for dissemination of tumor cells *via* blood circulation [9,10]. An additional mechanism that drives metastasis is the epithelial-to-mesenchymal transition (EMT), the process by which an epithelial cell type is transformed into a more migratory (*i.e.* metastatic) mesenchymal cell type [11,12]. The ECM plays an important role in EMT, as proteoglycans (PGs) modulate important EMT-promoting signaling pathways [13,14], such as the transforming growth factor beta (TGFβ) and interleukin-6 (IL-6) pathways [15,16], affect the expression of the anti-invasive, epithelial homotypic cell-adhesion molecule, E-cadherin [15,17] and mediate ECM remodeling and fibrillar alignment of the ECM in the peritumoral stroma [7,11].

Changes in the expression of the molecular players driving metastasis in general, and EMT specifically, include endocrine and ECM-dependent modes [11,18], but are also subjected to epigenetic regulation by microRNAs (miRNAs) [[19], [20], [21]]. miRNAs are endogenous non-coding RNA molecules, which regulate a wide range of physiological and pathological processes at the post-transcriptional level by inducing mRNA degradation in the RNA-induced silencing complex, and by translational repression [22,23].

The deregulation of miRNAs in breast cancer suggests a mechanistic contribution to the disease, which has been confirmed in numerous mechanistic studies, assigning either an oncogenic or a tumor-suppressive role to selected miRNAs [20,[24], [25], [26], [27]]. An important example of a miRNA serving as a tumor suppressor is miR-200b, which has been shown to regulate EMT in a variety of diseases, partially by targeting ZEB1 and ZEB2, two important transcriptional repressors of E-cadherin and by modulating TGFβ signaling [[28], [29], [30], [31]]. Notably, miR-200b deregulation has been linked to the resistance of breast cancer cells to the chemotherapeutic drugs doxorubicin and cisplatin, demonstrating the translational relevance of this miRNA for cancer therapeutic approaches [32,33].

We have previously shown that miRNA expression occurs in an ER-dependent manner in breast cancer and that altered expression of two ERβ-regulated miRNAs, miR-10b and miR-145, regulates the invasive phenotype and EMT program of breast cancer cells by targeting numerous ECM functional regulators [27]. We also reported that miR-200b expression is suppressed when ERα-positive MCF-7 breast cancer cells were cultured in estrogen-depleted medium, highlighting the significance of estradiol/ERα axis in the epigenetic regulation of this miRNA. In the present study, we further investigated the functional impact of ERα/β-dependent expression changes of the tumor suppressor miR-200b on ECM-related gene expression, EMT activation and the pathogenetic properties of ERα- and ERβ-positive mammary cancer cells. Our novel data demonstrate that depending on the presence of ERα or ERβ, targeting of the inversely regulated miR-200b, serves as a promising tool for the epigenetic control of aggressive breast cancer cells tumorigenic properties.

## Results

### ER status and signaling mediate miR-200b expression that is related to a better prognosis in breast cancer patients

**Fig. 1:**
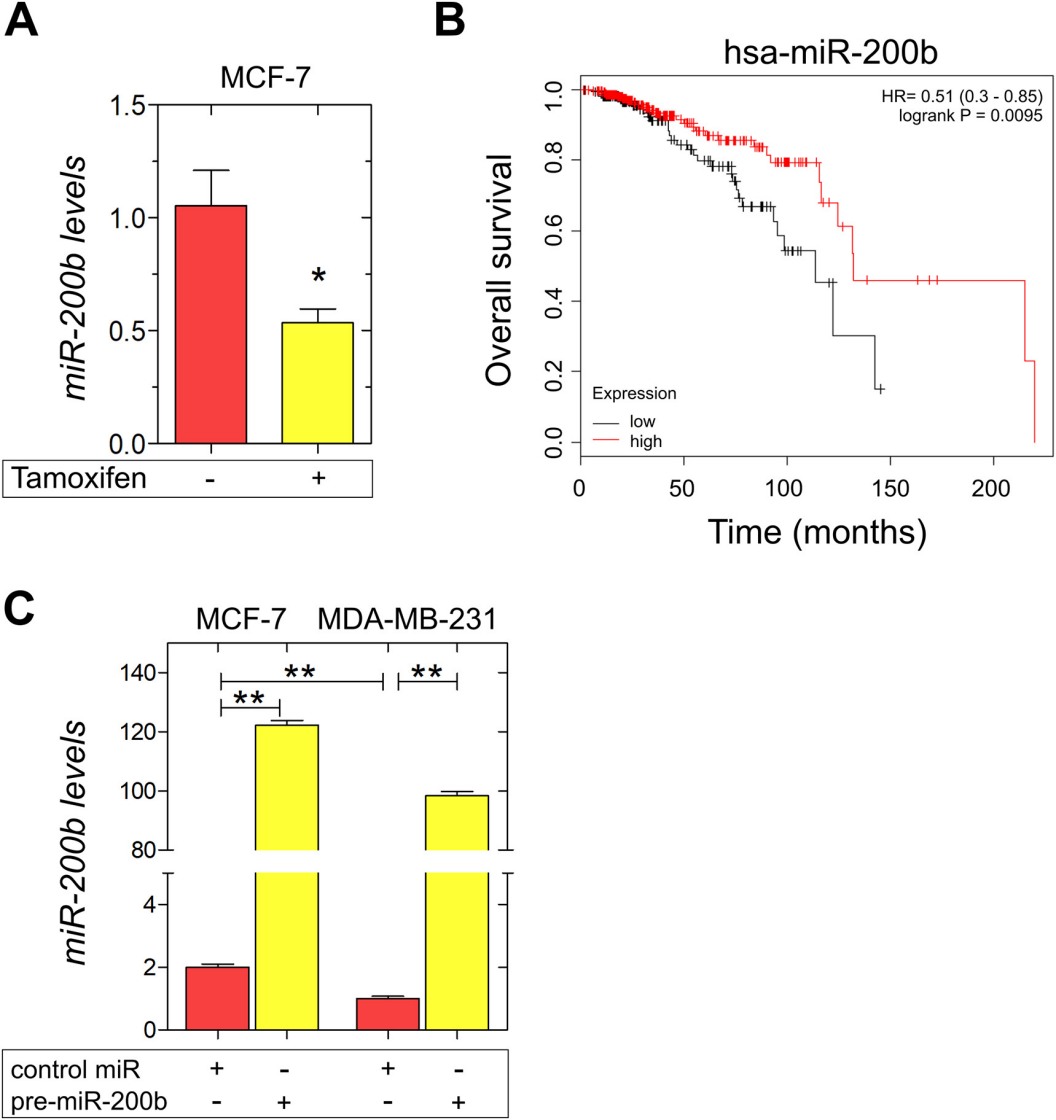
miR-200b expression is regulated by ER status and signaling and is correlated with better prognosis in breast cancer patients. (A) Tamoxifen treatment results in a downregulation of miR-200b expression in ERα-positive breast cancer cells, MCF-7. MCF-7 cells were treated with 100 nM tamoxifen for 24 h, followed by real-time qPCR analysis of miR-200b expression. (B) Kaplan-Meier survival analysis of human miR-200b (hsa-miR-200b) in breast cancer patients following systemic treatment. P value and hazard ratio (HR) value were calculated using a log-rank test [35]. (C) ΜCF-7 and MDA-MB-231 cells were transfected with a control miRNA (control miR) or a miR-200b precursor (pre-miR-200b) and the overexpression of miR-200b gene was monitored with real-time qPCR analysis. Expression was normalized to 18S rRNA expression. Asterisks (*), (**) indicate statistically significant differences (p ≤ 0.05 and p ≤ 0.01, respectively).

The expression of miRNAs is critical for the regulation of several cell functions including proliferation, migration, differentiation and survival [34]. Moreover, miRNAs are responsible for the direct regulation of EMT process and the expression of ECM components and ERs [20,27]. Deregulated miRNA expression is associated with normal processes, such as wound healing, as well as with pathological conditions, including cancer; however the impact of miRNAs in oncogenesis is still under debate [21,25,35]. Recent reports indicate that miR-200b restrains EMT through direct targeting of E-cadherin transcriptional repressors, ZEB1 and ZEB2, in breast cancer, thus modulating the disease progression [31,32,36]. Intriguingly We have previously shown that ER-mediated miR-200b expression is repressed when ERα-positive breast cancer cells are cultured in the absence of estrogen [27]. To evaluate the impact of blocking estrogen-dependent functions in a clinically relevant setting, we treated ERα-positive MCF-7 breast cancer cells with the SERM, tamoxifen [34]. Real-time qPCR analysis revealed that the inhibition of estrogen-dependent signaling resulted in a substantial and significant reduction of miR-200b expression (Fig. 1A). Kaplan-Meier survival analysis on a collective of 450 ER-positive breast cancer patients following systemic treatment [35] revealed that higher expression of human miR-200b (hsa-miR-200b) correlates with a better overall survival (Fig. 1B), suggesting the clinical importance of targeting miR-200b in mammary cancer. To note, the constitutive expression of miR-200b in the ERα-positive, epithelial cell line, MCF-7, is significantly higher compared to the mesenchymal, ERβ-positive, MDA-MB-231 breast cancer cell line (Fig. 1C), suggesting the crucial role of ER status in the regulation of miR-200b expression. This prompted us to further investigate the role of miR-200b in the regulation of breast cancer cell behavior following transfections with the precursor miR-200b (pre-miR-200b), which induces miR-200b overexpression (Fig. 1C).

### miR-200b inhibits the invasiveness and motility of MDA-MB-231 cells

**Fig. 2:**
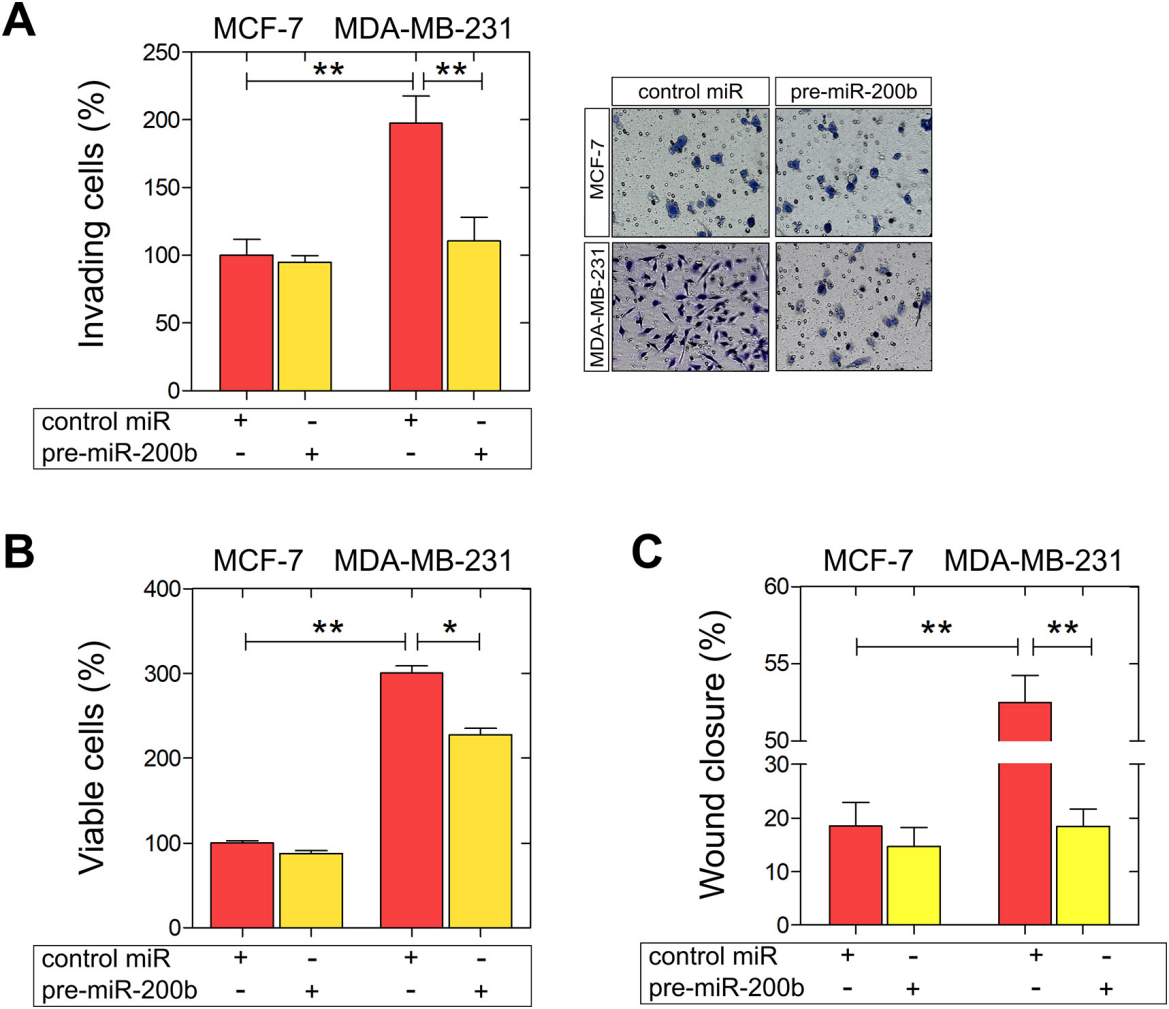
Functional analysis of miR-200b upregulation on invasiveness, viability and migration of MCF-7 and MDA-MB-231 breast cancer cells. Cells were transfected with a control miRNA (control miR) or pre-miR-200b. (A) miR-200b overexpression significantly inhibited MDA-MB-231 breast cancer cell invasiveness. Right panel: representative micrographs of invasion filter membranes after crystal violet staining. (B) miR-200b reduces cell growth and (C) migration after 24 h of ΜDA-MB-231 breast cancer cells. Asterisks (*), (**) indicate statistically significant differences (p ≤ 0.05 and p ≤ 0.01, respectively).

Decreased expression of miR-200 family is associated with lymph node metastasis in breast cancer [36]. Moreover, the aberrant silencing of miR-200b in breast cancer stem-like cells, which are largely responsible for cancer progression and metastasis, highlights the importance of epigenetic regulation in favour of breast cancer management [37]. This evidence prompted us to investigate the functional role of miR-200b in the modulation of breast cancer cells' pathogenetic properties. Using transient transfections with control miRNA, and pre-miR-200b, we evaluated the impact of miR-200b on invasiveness, cell viability and motility in a panel of two well-established breast cancer cell lines exhibiting different metastatic potential and ER status, MCF-7 (ERα-positive; low metastatic potential) and MDA-MB-231 (ERβ-positive; high metastatic potential). Our data revealed that in MDA-MB-231 cells, miR-200b upregulation significantly inhibited (*ca* 50%) the *in vitro* Matrigel invasiveness, as compared to the control miR-transfected MDA-MB-231 cells (Fig. 2A). Further, miR-200b upregulation decreased MDA-MB-231 cell growth (Fig. 2B). A strong decrease (*ca* 60%) in the migratory capacity of MDA-MB-231 cells overexpressing miR-200b was also observed, as compared to the control miR MDA-MB-231 cells (Fig. 2C). Intriguingly, the functional properties of the ERα-positive MCF-7 breast cancer cells were not considerably affected by miR-200b overexpression, underscoring the significance of the balance between miR-200b and ERα/β expression in the maintenance of breast cancer cell aggressiveness.

### miR-200b overexpression substantially alters cell morphology and regulates cytoskeletal structures in MDA-MB-231 cells

**Fig. 3:**
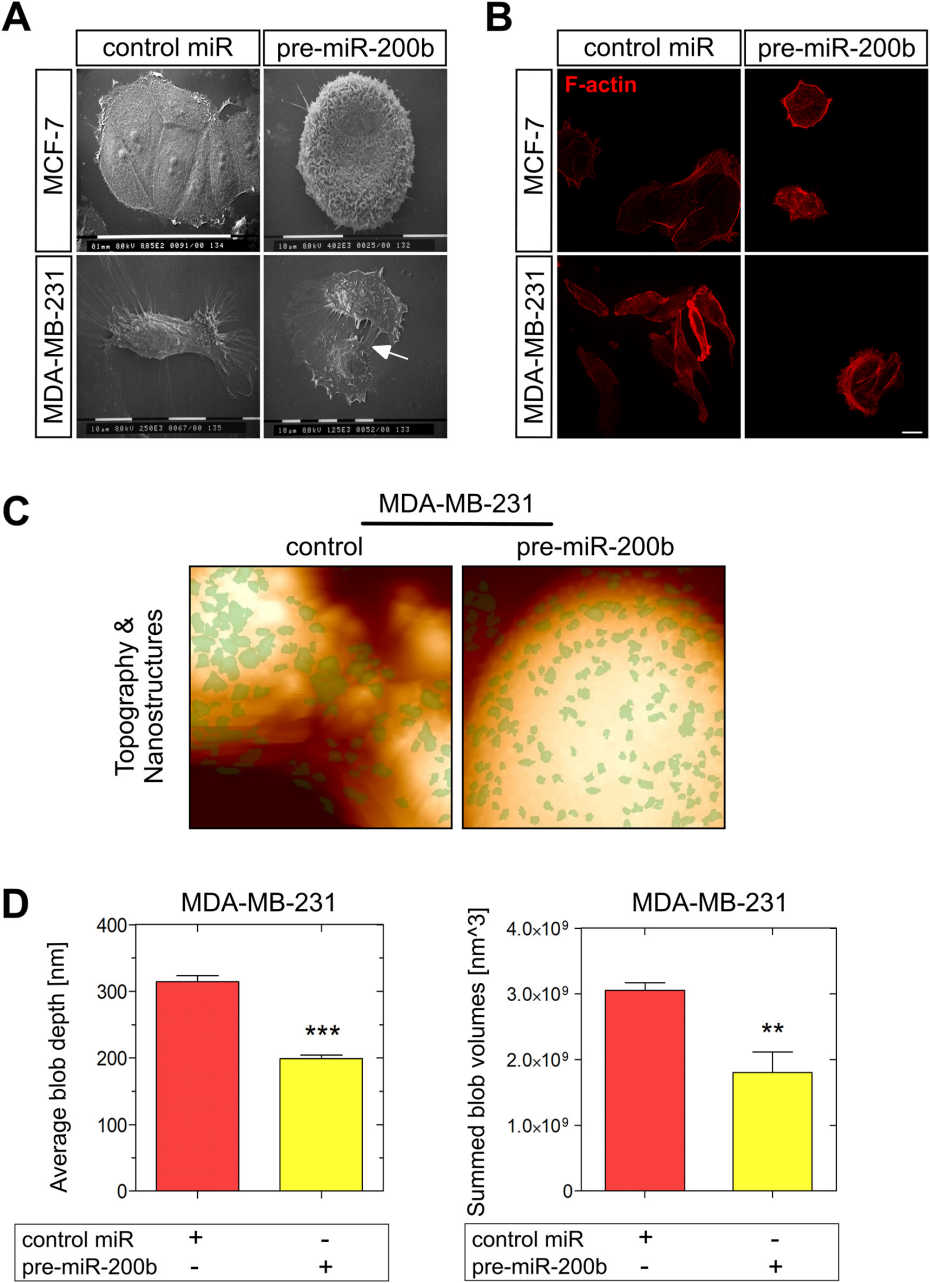
miR-200b overexpression results in altered morphology and regulates cytoskeletal structures in MDA-MB-231 breast cancer cells. (A) Cell morphology was monitored by SEM analysis. miR-200b overexpression resulted in less cytoplasmic protrusions and more cell-cell contacts (arrow) in ΜDA-MB-231 cells, whereas the morphology of MCF-7 cells was not affected by pre-miR-200b. (B) Immunofluorescence analysis of F-actin (red) in MCF-7 and ΜDA-MB-231 cells with or without pre-miR-200b (scale bar ~10 μm). (C, D) Nano-texture analysis. MDA-MB-231 cell surfaces transfected with control miR (left column) or with pre-miR-200b (right column) were imaged by AFM at nanometer resolution. Shown are the overlay of topography raw data of a 10 μm^2^ scan and protruding structure elements (green) (C), and (D) the number values for the object depth and the total volume [given as the sum of individual sizes (LDVs, local deviational volumes) per image]. Asterisks (**), (***) indicate statistically significant differences (p ≤ 0.01 and p ≤ 0.0001, respectively).

The invasive potential of breast cancer cells is largely related to their phenotype, which determines the interactions of cancer cells with matrix macromolecules of the surrounding tumor stroma, critical to mediate invasion and metastasis [7,36]. To further characterise the invasion-related phenotype of miR-200b depleted breast cancer cells, we employed a panel of complementary microscopic techniques. Using scanning electron microscope (SEM) we demonstrated that the induction of miR-200b expression in MDA-MB-231 breast cancer cells resulted in substantial morphological changes (Fig. 3A). Even though a more rounded morphology was observed in miR-200b-transfected MCF-7 cells, the epithelial phenotype was still retained (Fig. 3A). Notably, MDA-MB-231 cells transfected with pre-miR-200b lost the features of highly mobile cells, as their cytoplasmic protrusions were considerably less and they formed several cell-cell contacts, whereas the formation of pseudopodia (arm-like) and filopodia (thread-like) was more prominent in MDA-MB-231 cells in the absence of pre-miR-200b. These observations were followed by clear changes in cell cytoskeleton (F-actin staining) of MDA-MB-231 cells (Fig. 3B). The F-actin staining for cytoskeleton revealed a more condensed cytoskeletal network in MDA-MB-231 cells transfected with pre-miR-200b, in comparison with the characteristic mesenchymal-like characteristics in the cytoskeleton formation of control miR MDA-MB-231 cells. As we hypothesized that miR-200b-dependent regulation of MDA-MB-231 cell morphological characteristics may be involved in their less invasive phenotype, we preformed further morphological investigations using atomic force microscopy (AFM). Typical topographic images obtained by nanotexture analysis demonstrated smoother surface of MDA-MB-231 cells transfected with pre-miR-200b compared to the fine ruffles observed in control cells (Fig. 3C). The number and distribution of objects among control and pre-miR-200b-treated MDA-MB-231 cells did not exhibit a specific pattern (green spots), however, the average blob depth is strongly decreased from 314 to 198 nm, respectively, as the sum of object volumes was significantly reduced accordingly from 3 × 10^9^ to 1.8 × 10^9^ nm^3^ (Fig. 3D). The significant reduction in depth and volume of cytoskeletal structures on MDA-MB-231 cells with pre-miR-200b confirms the strong cytoskeletal rearrangement observed.

### miR-200b attenuates the EMT program in MDA-MB-231 cells

**Fig. 4:**
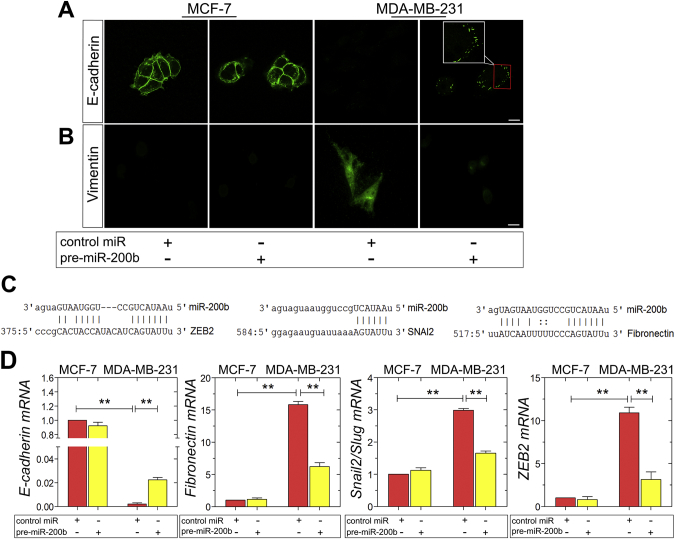
The overexpression of miR-200b inhibits in EMT process in MDA-MB-231 breast cancer cells. (A, B) Immunofluorescence analysis of E-cadherin (green) and vimentin (green) in MCF-7 and ΜDA-MB-231 cells with or without pre-miR-200b (scale bar ~10 μm). (C) Fibronectin, Snail2/Slug and ZEB2 are predicted targets of miR-200b. An alignment of the miR-200b seed sequence with the 3′UTRs of these mRNAs was performed utilizing the public target prediction database microRNA.org. Number indicate the corresponding base pairs in the target mRNA sequence. (D) mRNA Expression of the epithelial marker E-cadherin and the mesenchymal markers fibronectin, Snail2/Slug and ZEB2 in MCF-7 and ΜDA-MB-231 cells with or without pre-miR-200b, as determined by qPCR. Asterisks (*), (**) indicate statistically significant differences (p ≤ 0.05 and p ≤ 0.01, respectively).

The conversion of early stage tumors into invasive malignancies is a hallmark in tumorigenesis and is activated by the fundamental EMT program. It has been demonstrated that miR-200b upregulates E-cadherin through direct targeting of its transcriptional repressors, ZEB1 and ZEB2, thus inhibiting EMT [28,32,36]. Moreover, it is reported that the autocrine ZEB2/miR-200b feedback loop regulates the establishment and maintenance of EMT [37,38]. The observed morphological changes in MDA-MB-231 breast cancer cells as well as in their functional properties, resulting by miR-200b upregulation, generated the question whether miR-200b is also capable of modulating the EMT program in these mesenchymal cells. Confocal microscopy of E-cadherin staining revealed the typical adherens junctions in MCF-7 cells, which are not affected by pre-miR-200b (Fig. 4A). Intriguingly, the characteristic E-cadherin spots reappeared in MDA-MB-231 cells following miR-200b upregulation (Fig. 4A). Further, MDA-MB-231 cells overexpressing miR-200b exhibited significantly reduced protein levels of the mesenchymal marker vimentin, as compared to control miR MDA-MB-231 cells (Fig. 4B). *In silico* analysis of EMT-related markers as predicted targets of miR-200b was performed employing the public database microRNA.org. The EMT regulators ZEB2, Snail2/Slug, and the mesenchymal marker and interstitial matrix protein fibronectin were identified as predicted targets (Fig. 4C). The EMT reprogramming caused by pre-miR-200b in MDA-MB-231 cells was confirmed by evaluating the mRNA levels of major EMT these EMT markers, and of the ZEB2-mediated epithelial marker E-cadherin, by real-time qPCR. Specifically, in accordance with the target prediction, pre-miR-200b-transfected MDA-MB-231 cells demonstrated a considerable increase in E-cadherin levels by 4-fold, accompanied by substantially decreased mRNA levels of the mesenchymal markers fibronectin, Snail2/Slug and ZEB2, by 10-, 1.5- and 7.5-fold, respectively (Fig. 4D). Collectively, these data suggest that miR-200b is a key switch of EMT in the aggressive MDA-MB-231 breast cancer cells, since miR-200b overexpression reverses EMT in these cells.

### miR-200b overexpression affects matrix composition and signaling in MDA-MB-231 cells

**Fig. 5:**
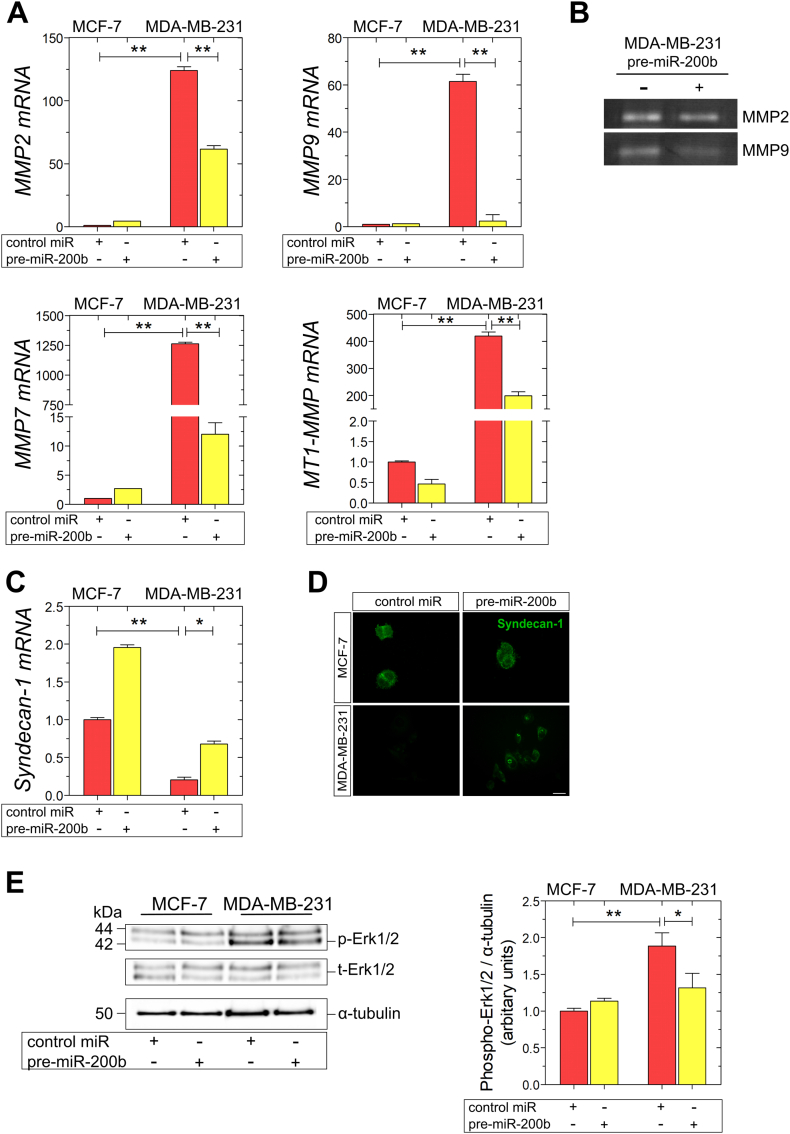
miR-200b regulates matrix composition and signaling in MDA-MB-231 breast cancer cells. Real-time qPCR analysis of major MMPs (MMP2, MMP7, MMP9, MT1-MMP) (A), (B) MMP2/MMP9 gelatinolytic activities (as assayed by gelatin zymography), and expression (mRNA and protein) of the cell membrane PG, syndecan-1 (C, D), following miR-200b overexpression in MCF-7 and ΜDA-MB-231 cells with real-time qPCR analysis and immunofluorescence microscopy. The mRNA levels were evaluated using β-actin as reference gene. (E) Immunoblots of phospho-Erk1/2, total-Erk1/2 and α-tubulin in MCF-7 and ΜDA-MB-231 cells in the presence or absence of pre-miR-200b. Asterisks (*), (**) indicate statistically significant differences (p ≤ 0.05 and p ≤ 0.01, respectively).

As demonstrated above, miR-200b is a key switch of EMT, however, its role in controlling matrix composition in breast cancer cells still remains a challenge. Based on our analysis of EMT markers, we hypothesized that the switch of the cellular phenotype towards a more epithelial morphology and the downregulation of the interstitial matrix constituent fibronectin may affect the expression and function of additional ECM proteins. As far as the regulation of proteolytic enzymes in MDA-MB-231 cells is concerned, miR-200b overexpression strongly reduced the expression levels of MMP2, MMP7, MMP9 and MT1-MMP (Fig. 4C). Moreover, it has been recently reported that ERs regulate the expression of syndecan-1 in endocrine-associated breast cancer [36]. Regarding the effect of miR-200b on cell membrane PGs, pre-miR-200b resulted in the strong increase of syndecan-1 mRNA and protein levels in MDA-MB-231 cells in comparison with control miR MDA-MB-231 cells (Fig. 5B and C). As it is depicted in Fig. 5D, pre-miR-200b transfection resulted in the significant decrease of the phosphorylated Erk1/2 forms in MDA-MB-231 cells, compared to control miR MDA-MB-231 cells. In summary, these results clearly demonstrate that miR-200b is strongly implicated in the regulation of ECM composition and signaling in MDA-MB-231 cells, following the inhibition of their metastatic potential through miR-200b upregulation.

## Discussion

Accumulating evidence suggests that gene expression in diverse signaling pathways is post-transcriptionally regulated by the epigenetic mechanism of miRNAs [37]. The deregulated miRNA expression has been associated with normal and pathological conditions, such as cancer [20]. Among the deregulated miRNAs, miR-200 family has been studied in several cancer types including breast cancer [31]. miR-200b serves as a pluripotent miRNA with important roles in regulating breast cancer aggressiveness, highlighting its prominent diagnostic and therapeutic potential in breast cancer. Low miR-200b expression levels coincide with the generation of metastatic breast cancer stem-like cells [38] and poor survival in breast cancer. Our study extends these findings to the subgroup of ER-positive systemically treated breast cancer patients, where a high expression of miR-200b was associated with a better survival. miR-200b has furthermore been linked to the mesenchymal characteristics of mammary carcinoma cells acting as a negative regulator of tumor invasion and metastasis [28]. Reduced expression of miR-200 family members contributes to resistance to antiestrogenic treatment in breast cancer cells [39], which may explain our observation of tamoxifen-induced downregulation of miR-200b as a possible resistance mechanism. Further, the double negative feedback loop among miR-200b, TGFβ and ZEB2 transcription factor modulates the establishment of EMT [[40], [41], [42]]. Intriguingly, the 3′ untranslated regions of ERα and ERβ genes belong to the direct targets of miR-200b [43], suggesting the clinical significance of targeting this miRNA in breast cancer. In this study, we pinpointed the functional link between ERβ and miR-200b expression in MDA-MB-231 breast cancer cells. We demonstrated that the presence of ERβ in highly invasive MDA-MB-231 cells is responsible for maintaining low miR-200b constitutive expression, as compared to the high miR-200b levels in the ERα-positive MCF-7 breast cancer cells (low invasive potential). We demonstrated for the first time the regulatory role of this miRNA in the modulation of aggressive breast cancer cell behavior in the context of EMT activation and matrix composition, suggesting its prominent aspect in breast cancer progression.

Functional analysis revealed that miR-200b overexpression resulted in considerable inhibition if the invasive and migratory capacity of MDA-MB-231 breast cancer cells. Moreover, miR-200b slightly reduced the growth rates of these cells, as compared to the control ones. Of note, the metastatic potential of MCF-7 epithelial cells was not substantially affected by miR-200b upregulation apparently due to the constitutively high levels of this miRNA, which is a key factor for the low aggressiveness of these cells, as previously reported [27,44]. While MCF-7 cells already exhibit an epithelial-like phenotype, the miR-200b-induced switch towards a more epithelial phenotype was particularly effective in alleviating invasive properties in the basal-like breast cancer cell line MDA-MB-231, which exhibits a mesenchymal, highly migratory and invasive phenotype.

The induction of EMT is critically regulated by the tight interaction of cancer cells with their surrounding ECM [8]. Considering the high specialization of ECM as scaffold composed of a variety of functional macromolecules interacting with each other to mediate physiological functions of cells within tissues, such as cell proliferation, survival, migration, and differentiation, thus maintaining tissue functions and homeostasis. Notably, ECM molecules interact with resident cells of each tissue through numerous cell surface receptors to mediate vital cell responses, such as cell adhesion, differentiation and migration, cytoskeleton conformation and cell signaling [[45], [46], [47]]. In breast cancer, ERs and ECM mediators, such as MMPs, PGs and GAGs, exert a prominent role in the activation and maintenance of this process, thus affecting cancer progression [[48], [49], [50], [51]]. We have previously demonstrated that miR-200b acts synergistically with the estradiol-ERα axis to conserve the epithelial profile of MCF-7 breast cancer cells. On the other hand, the estradiol-ERβ pathway in MDA-MB-231 is the critical factor for the maintenance of the low expression levels of miR-200b in these cells and that ERβ suppression induces miR-200b expression, characterizing cells with a less aggressive phenotype [27]. The data of this study initially confirmed that the presence of miR-200b failed to activate EMT program in ERβ-positive MDA-MB-231 cells as it was observed by the strong reduce in the expression levels of ZEB2, which is the predicted target of miR-200b [52,53], and major changes in protein and mRNA levels of important mesenchymal and epithelial markers. This observation was followed by substantial changes in MDA-MB-231 ultrastructure, cytoskeleton organization and the distribution of cytoskeletal structures in cell surface, confirming that miR-200b overexpression weakens the mesenchymal phenotype and the invasive capacity of aggressive breast cancer cells. The overexpression of miR-200b in MDA-MB-231 cells resulted in a significant reduce in the expression levels of major proteolytic enzymes that mediate ECM remodeling and promote cancer progression [[54], [55], [56]], including MMP2, MMP7, MMP9 and MT1-MMP. Moreover, miR-200b promotes the expression of syndecan-1, which has been previously correlated with the reduced cell migration and strong adhesion. In line with our findings, including the downregulation of the integrin ligand fibronectin, a miR-200b-associated change in metastatic breast cancer cell behavior has been linked to the integrin-regulatory focal adhesion compound kindlin-2, which is by itself a target of miR-200b, providing an additional functional link between cell-matrix interplay and miR-200b-dependent changes in EMT [57,58]. In our study, the changes in ECM composition were associated with the a significant decrease in the phosphorylated levels of Erk1/2 kinases, which have been correlated with breast cancer cell proliferation and migration [59,60], suggesting the critical role of miR-200b in the activation of this signaling pathway in ERβ-positive MDA-MB-231 breast cancer cells. In conclusion, taking into consideration the above data, we underscored the importance of ERβ as an epigenetic mediator of miR-200b in aggressive breast cancer cells. This novel data highlights the significance of targeting the ERβ-mediated miR-200b as a promising theranostic tool for the management of aggressive mammary carcinoma.

## Materials and methods

### Chemicals, reagents and antibodies

Dulbecco's modified eagle medium (DMEM), fetal calf serum (FCS), l-glutamine, penicillin, streptomycin were all obtained from Gibco BRL (Karlsruhe, Germany). All other chemicals used were of the best commercially available grade. Antibodies used are listed in Supplementary Table 1.

### Cell culture and miRNA transfections

MDA-MB-231 (high metastatic potential, ERα-negative, ERβ-positive) and MCF-7 (low metastatic potential, ERα-positive) breast cancer cell lines were obtained from the American Type Culture Collection (ATCC) and routinely cultured as monolayers at 37 °C in a humidified atmosphere of 5% (v/v) CO_2_ and 95% air. Breast cancer cells were cultured in DMEM supplemented with 10% (v/v) FCS, 1% l-glutamine and 1% penicillin/streptomycin. Cells were harvested by trypsinization with 0.05% (w/v) trypsin in PBS containing 0.02% (w/v) Na_2_EDTA. All experiments were conducted in serum-free conditions. Transfections of MCF-7 and ΜDA-MB-231 breast cancer cells with the miRNA precursor pre-miR-200b (10 nM, Applied Biosystems), or miRNA mimic, negative control #1 (10 nM, Applied Biosystems) were performed according to the manufacturer's instructions and as described previously [61]. The transfection reagent for the performed experiments was the DharmaFECT (Dharmacon, GE Healthcare), which was used according to the manufacturer's instructions. The overexpression of miR-200b was confirmed by real-time qPCR analysis.

### RNA isolation, reverse transcription and real-time qPCR analysis

Total cellular RNA was isolated using rna-OLS (OMNI Life Science, Hamburg, Germany) and reverse transcribed (Advantage First strand cDNA synthesis kit; Fermentas, St. Leon-Rot, Germany). For miRNA isolation and analysis, the mirVanaTM miRNA Isolation Kit (Applied Biosystems) and the TaqManVR MicroRNA RT kit (Applied Biosystems) were employed. Real-time qPCR and melting curve analysis were performed using Qiagen QuantiTect SYBR Green PCR kit in a LightCycler (Roche, IN). The expression of miRNAs and additional mRNAs was analyzed using TaqMan probes on an ABI PRISM 7300 Sequence Detection System as described previously [61]. The 2^−ΔΔCt^ method was used to determine relative gene transcript levels following normalization to 18S rRNA. Primer sequences, QuantiTect assay and TaqMan probe IDs are listed in Supplementary Tables 2 and 3.

### Tamoxifen treatment

2 × 10^5^ MCF-7 cells/well of a 12 well-plate were treated with 100 nM tamoxifen (Sigma-Aldrich) or vehicle (ethanol) for 24 h, followed by preparation of RNA lysates and RNA isolation using the innuPREP RNA Mini Kit (Analytik Jena AG, Germany) according to the manufacturer's instructions. miRNA was converted into cDNA using the TaqMan MicroRNA Reverse Transcription Kit (Applied Biosystems). Expression of miR-200b with the TaqMan probe hsa-miR-200b (Applied Biosystems, Darmstadt, Germany) was detected by qPCR as previously described [27] using the small RNA RNU44 (Applied Biosystems) for normalization.

### Cell proliferation assay

MTT [3-(4,5-dimethylthiazol-2-yl)-2,5-diphenyltetrazolium bromide] assay was carried out for 24 h as previously described [62]. 72 h after miRNA transfection, 10^4^ MCF-7 and MDA-MB-231 cells were seeded in 96-well plates and cultured for 24 h, followed by 24 h incubation in the presence of MTT, lysis and optical density measurement at 595 nm in a microplate reader.

### *In vitro* cell invasion assay

2.5 × 10^4^ transfected breast cancer cells in 0.5 mL DMEM/10% FCS were added in triplicates to the upper compartments of Matrigel Invasion Chambers (BD Biosciences, Heidelberg, Germany) 48 h after miRNA transfections. After 24 h, the medium in the upper chamber was replaced by serum-free DMEM. After 48 h, cells on the lower surface were fixed and stained with DiffQuik (Medion, Duedingen, Switzerland). Relative invasiveness was expressed as percentage of cells on compound-treated compared to control inserts (n > 3).

### *In vitro* wound healing assay

2.5 × 10^4^ transfected breast cancer cells per well were seeded in 12-well cell culture plates. Confluent cell layers were serum-starved for 16 h and then wounded by scratching with a sterile 100 μL pipette tip. Detached cells were removed by washing twice with PBS and fresh culture medium, in the absence of serum, was added. The wound closure was monitored at 0, 16 and 24 h using a digital camera connected to a microscope. Wound surface area was quantified by image analysis (Image J software).

### SEM imaging

Breast cancer cells seeded in culture flasks 48 h after miRNA transfections were fixed in a Karnovsky's solution for 20 min. Flasks with adhering cells were again rinsed three times with 0.1% cacodylate buffer, dehydrated with increasing concentrations of ethanol, and finally dehydrated with hexamethyldisilazane (Sigma-Aldrich Inc.) for 15 min. The specimens were mounted on appropriate stubs, coated with a 5 nm palladium gold film (Emitech 550 sputter-coater) to be observed under a SEM (Philips 515, Eindhoven, The Netherlands) operating in secondary-electron mode.

### Confocal immunofluorescence microscopy

5 × 10^4^ transfected breast cancer cells were seeded onto coverslips in 24-well plate and incubated in DMEM containing 10% FCS for 24 h, fixed in 4% paraformaldehyde in PBS and permeabilized with PBS/0.1% TritonX-100. Nonspecific binding was blocked with PBS/1% Aurion BSA-c (DAKO, Glostrup, Denmark). Coverslips were subsequently incubated with the primary antibodies at 4 °C overnight and incubated with Alexa Fluor-conjugated antibodies for 30 min at room temperature. Primary antibody omission served as a negative control. Slides were analyzed with the LSM 510 META confocal microscope equipped with the oil immersion objective Plan-Apochromat 63x/1.40 (Carl Zeiss, Jena, Germany). Representative images were analyzed by image analysis (ZEN Software).

### Surface nano-texture analysis (nAnostic)

Contact mode Atomic Force Microscopy (AFM) on cultivated cells was performed as described before [63]. In this study, cells were chemically stabilized by glutardialdehyde fixation (1% final concentration). Briefly, AFM measurements were carried out in PBS-buffered solution (pH 7.4) using a Multimode AFM equipped with Nanoscope III controller and software version 5.30 sr3 (Digital Instruments, Santa Barbara, CA, USA). Silicon-nitride tips on V-shaped gold-coated cantilevers were used (0.01 N/m, MLCT, VEECO, Mannheim, Germany). Imaging was performed at ambient temperature with forces <1 nN at 1–3 scan lines per second (1–3 Hz) with 512*512 pixels resolution. For texture analysis, subcellular scan areas of 10μm^2^ are recorded. Topographical data of the cell surfaces were analyzed using the nAnostic™-method applying custom-built, proprietary algorithms (Serend-ip GmbH, Münster, Germany). The method principle has been described before [64]. Briefly, each nanostructure protruding from the mean surface level is morphometrically evaluated. Then, they are filtered by their size and shape through computer vision; here, only structures of positive local deviational volume (LDV), smaller than 10^3^ nm in height and an area smaller than 10^6^ nm were considered. Values are given for the average depth of such objects (per image) and the sum of their deviational volumes (LDVs).

### Western blot analysis

Cell lysates of breast cancer cells were prepared 72 h after transfection with control or miRNAs. Cell lysates were prepared using modified radioimmunoprecipitation buffer with proteinase inhibitors [59]. Cell lysates were separated on 10% SDS-PAGE, the proteins were electrophoretically transferred to PVDF membranes (Bio-Rad, USA) and blotted with the indicated antibodies as described previously [59], using 30–60 μg of protein/lane.

### Kaplan Meier plotter survival analysis

Kaplan Meier survival analysis was performed using the online tool mirPower [52]. Details on the patient collective have been previously published in reference [52]. miR-200b mRNA expression data from the TCGA dataset of a collective of 450 ER-positive systemically treated breast cancer patients were stratified by high and low expression using the mirPower default settings (cutoff value 463, expression range 2–2341) and correlated with overall survival.

### Statistical analysis

Reported values are expressed as mean ± standard deviation (SD) of experiments in triplicate. Statistically significant differences were evaluated using the analysis of variance (ANOVA) test and were considered statistically significant at the level of at least p ≤ 0.05. Statistical analysis and graphs were made using GraphPad Prism 6 (GraphPad Software). Kaplan Meier survival data were analyzed using the mirPOWER online tool as described in reference [52]. The package “survival” of the R statistical environment was used to calculate hazard ratio (HR), 95% confidence intervals (CI), and log-rank p-values.

## Funding information

This work has received funding by the EU Horizon 2020 project RISE-2014, action No. 645756 “GLYCANC – Matrix glycans as multifunctional pathogenesis factors and therapeutic targets in cancer”. Z.P. was supported by the 10.13039/501100001655DAAD agency, grant No. 91607321.

## Author contributions

Z.P. performed the main experimental part and prepared the manuscript draft and the figures and submitted the manuscript. M.F. performed SEM image analysis and interpretation, C.R. contributed in AFM and analyzed the data. M.G. and N.K.K. had the overall supervision of the experiments, demonstration of the data and contributed to manuscript writing and editing. All authors reviewed the manuscript.

## Declaration of competing interest

C.R. is an employee of Serend-ip GmbH; the other authors declare no conflict of interest.

## Appendix A Supplementary data

Supplementary tables Image 1
