# Supplementary material for: miR-200b restrains EMT and aggressiveness and regulates matrix composition depending on ER status and signaling in mammary cancer
Source: Matrix Biol Plus. 2020 Jan 22;6-7:100024. doi: 10.1016/j.mbplus.2020.100024 (PMC7852204; doi:10.1016/j.mbplus.2020.100024)
Supplement: Supplementary file 1 — Supplementary tables [file mmc1.docx]

**miR-200b restrains EMT and aggressiveness and regulates matrix composition depending on ER status and signaling in mammary cancer**

Zoi Piperigkou, Marco Franchi, Christoph Riethmüller, Martin Götte, Nikos K. Karamanos

**Supplementary material**

Supplementary Table 1. List of antibodies used in this study.

| **Antibody** | **Manufacturer** |
| --- | --- |
| Alexa-Fluor 488 anti-rabbit IgG, Goat | Invitrogen Corporation, Carlsbad, USA |
| Alexa-Fluor 488 anti-mouse IgG, Goat | Invitrogen Corporation, Carlsbad, USA |
| Alexa-Fluor 568- labeled phalloidin | Invitrogen Corporation, Carlsbad, USA |
| anti-human E-Cadherin, clone 36, monoclonal, Mouse | BD, Becton & Dickinson Biosciences, Heidelberg, Germany |
| anti-human Vimentin, clone 13.2, monoclonal, Mouse | Sigma-Aldrich, Steinheim, Germany |
| anti-human-phospho -p44/42 MAPK (Erk1/2) (Thr202/Tyr204**)**, polyclonal, Rabbit | Cell Signaling Technology, Inc., Danvers, USA |
| anti-human-Syndecan-1 (DL-101), monoclonal Mouse | Santa Cruz Biotechnology Inc., Santa Cruz, USA |
| anti-human-α-Tubulin, clone B-5-1-2, Mouse | Sigma-Aldrich, Steinheim, Germany |
| goat anti-rabbit IgG, Peroxidase Conjugated | Merck Millipore, Germany |
| goat anti-mouse IgG, Peroxidase Conjugated | Merck Millipore, Germany |

Supplementary Table 2. Real-time qPCR assay used in the validation phase of the study.

| **miRBase ID** | **miRBase Accession No.** | **Mature miRNA sequence** |
| --- | --- | --- |
| hsa-miR-200b | MIMAT0000318 | UAAUACUGCCUGGUAAUGAUGA |

Supplementary Table 3. List of real-time qPCR primers used in this study.

| **Gene** | **Primer code / sequence** | **Primer type** |
| --- | --- | --- |
| 18S rRNA | Hs99999901_s1 | ABI TaqMan assay |
| Actin gamma-2 | Hs01123712_m1 | ABI TaqMan assay |
| E-cadherin (CDH1) | Hs00170423_m1 | ABI TaqMan assay |
| ZEB2 | Fwd: 5’- TGGGCTAGTAGGCTGTGTCCA-3’  Rev: 5’- TCATCTTCAACCCTGAAACAGAGG-3’ | conventional PCR primer |
| Fibronectin | Fwd: 5’-CCAAGCATCACCCTGGGAGT-3’  Rev: 5’-CGAAGCAGAACAGGCAATGTG-3’ | conventional PCR primer |
| Snail2/Slug | Fwd: 5’-ATCTGCCAGACGCGAACTCA-3’  Rev: 5’-GGCAACCAGACAACCGACAT-3’ | conventional PCR primer |
| MMP2 | Hs00234422_m1 | ABI TaqMan assay |
| MMP7 | Fwd: 5’-GCTGGCTCATGCCTTTGC-3’  Rev: 5’-TCCTCATCGAAGTGAGCATCTC-3’ | conventional PCR primer |
| MMP9 | Hs00234579_m1 | ABI TaqMan assay |
| MT1-MMP | Fwd: 5’-CATGGGCAGCGATGAAGTCT-3’  Rev: 5’-CCAGTATTTGTTCCCCTTGTAGAAGTA-3’ | conventional PCR primer |
| Syndecan-1 | Hs00174579_m1 | ABI TaqMan assay |
